# Prevalence of Carotid Atherosclerotic Plaques and Stenosis in Adults with Familial Hypercholesterolemia Needs Reappraisal: Systematic Review and Meta-Analysis

**DOI:** 10.3390/jcm14248676

**Published:** 2025-12-07

**Authors:** Marcin Piechocki, Magdalena Kaźnica-Wiatr, Anna Kabłak-Ziembicka

**Affiliations:** 1 Doctoral School of Medical and Health Sciences, Jagiellonian University Medical College, ul. św. Łazarza 16, 31-530 Kraków, Poland; marcin.piechocki@doctoral.uj.edu.pl; 2 Department of Radiology and Diagnostic Imaging, St. John Paul II Hospital, Prądnicka 80, 31-202 Kraków, Poland; 3 Department of Cardiac and Vascular Diseases, St. John Paul II Hospital, Prądnicka 80, 31-202 Kraków, Poland; mkaznica@mp.pl; 4 Center for Innovative Medical Education, Department of Medical Education, Faculty of Medicine, Jagiellonian University Medical College, 31-008 Kraków, Poland; 5 Department of Cardiac and Vascular Diseases, Institute of Cardiology, Jagiellonian University Medical College, św. Anny 12, 31-008 Kraków, Poland

**Keywords:** familial hypercholesterolemia, carotid plaques, carotid stenosis, cardiovascular risk, premature atherosclerosis, systematic review, meta-analysis

## Abstract

**Background:** This systematic review and meta-analysis aimed to assess the burden of carotid atherosclerosis, including the prevalence of carotid plaques and stenosis, among individuals with familial hypercholesterolemia (FH). **Methods:** PubMed, Embase, and Scopus were searched up to 3 August 2025 to identify full-text, peer-reviewed articles published in English reporting the prevalence of carotid atherosclerotic plaques and/or stenosis in adult (≥18 years) patients with either a clinical or genetic diagnosis of FH. The methodological quality of the included studies was assessed using the Mixed Methods Appraisal Tool. Results were synthesized using random-effects meta-analysis and presented as pooled prevalence estimates with 95% confidence intervals (95% CI) displayed in forest plots. Publication bias was assessed using the Doi plot and the Luis Furuya-Kanamori index. **Results:** For the analysis of carotid plaque prevalence, seventeen studies including a total of 2870 patients were included (weighted age 47.2 ± 13.4 years, 47.3% male). No statistical difference in the pooled prevalence of carotid plaques was observed between clinically and genetically diagnosed FH (both 53%; 95% CI: 40–65%), however sub-analyses suggested a higher plaque burden in genetic FH. For the analysis of carotid stenosis prevalence, four studies comprising 704 participants were included; however, the available data were less consistent, yielding a pooled prevalence of 9% (95% CI: 0–40%). In conclusion, the results should be interpreted with caution due to several limitations, including the relatively low quality of the included studies, potential publication bias, considerable heterogeneity between the studies, and low to moderate certainty of evidence for the pooled estimates. These findings further emphasize the need for large-scale, standardized, multicenter studies to better characterize the burden of carotid atherosclerosis in this population.

## 1. Introduction

Familial hypercholesterolemia (FH), the most common genetic lipid disorder, is characterized by markedly elevated levels of low-density lipoprotein cholesterol (LDL-C) since birth [1]. The more prevalent heterozygous form of FH affects approximately 1 in 300 individuals worldwide and is typically associated with naive LDL-C concentrations ranging from 190 to 400 mg/dL [2,3]. Homozygous FH, historically estimated at 1 case per million population, is observed to be more prevalent in recent studies, accounting for 1 case per 170,000 to 300,000 [4]. Homozygotes of FH may have plasma LDL-C levels that are at least twice as high as those of heterozygous FH and therefore several times higher than normal serum levels [5].

FH leads to a substantially increased risk of premature atherosclerotic cardiovascular disease (ASCVD) [5,6]. In particular, FH stimulates progression of atherosclerotic lesions in coronary and lower limb artery territories [6,7,8]. Most studies assessing the cardiovascular burden in FH have focused on coronary artery disease (CAD), where the risk is estimated to be up to 13-fold higher than in the general population, affecting roughly one-third of patients with FH [8,9,10,11,12,13,14]. It has been reported that 50% of men by the age of 50 years and up to 30% of women by the age of 60 years will experience myocardial infarction (MI) when heterozygous FH remains untreated [12]. Homozygous FH imposes extremely high risk of major adverse cardiac and cerebral events (MACCE) [14]. Fatal cardiovascular complications could develop even in the first decade of life [4]. The coronary threshold would be around 11 years old even for individuals with lower LDL-C levels [8]. Most patients would not survive beyond 30 years of age when left untreated [15].

Data on the prevalence of peripheral arterial disease (PAD) in FH are less consistent, with reported rates ranging from as low as 0.3% to as high as 60%, depending on the study population and diagnostic methods applied [16]. Nevertheless, FH has been associated with up to threefold increased risk of PAD compared to the general population [17]. Data for renal artery stenosis in FH are not available. Emanuelsson et al. reported odds ratios (ORs) for PAD in individuals with FH as: 1.84 (95% CI: 1.70–2.00) in those with possible FH and 1.36 (95% CI: 1.00- 1.84) in individuals with probable/definite FH, whereas respective ORs of having chronic kidney disease were 1.92 (95% CI, 1.78–2.07) and 2.42 (95% CI, 1.86–3.26) [18]. In line, compared with individuals with unlikely FH and Ankle-Brachial Index (ABI) above 0.9, the multivariable adjusted hazard ratio (95% CI) of MI was 4.60 (95% CI, 2.36–8.97) in those with possible/probable/definite FH and ABI below 0.9 [18].

The only reliable method to confirm FH is genetic testing [19]. The most common genetic disorders altering the metabolism of LDL-C include mutations in the gene for LDL receptor (LDLR), and less commonly in those for apolipoprotein B (APOB), proprotein convertase subtilisin-kexin type 9 (PCSK9), and others [1,19,20].

Although, genetic testing remains the gold standard for confirming the diagnosis of FH [1,19,20], several algorithms for FH diagnosis were built to facilitate patient management [21,22,23,24,25,26,27]. These algorithms include not only the magnitude of the naive LDL-C levels, but also a personal and family history of premature ASCVD, and physical findings such as tendon xanthomas and arcus corneae. Various combinations of these clinical features are incorporated into diagnostic algorithms used to estimate the probability of FH [21,22,23,24,25,26,27]. Among these, the Dutch Lipid Clinic Network (DLCN) criteria are the most widely used and validated [23]. Other established diagnostic approaches include Simon Broome criteria, Welsh FH score, Make Early Diagnosis to Prevent Early Deaths (MEDPED), as well as variety of national guidelines, such as Japanese Atherosclerosis Society guidelines criteria or Canadian FH criteria [24,25,26,27].

By contrast, evidence regarding atherosclerotic involvement of the carotid arteries in FH remains limited [28]. In the general population, carotid atherosclerosis is observed in approximately 20% of individuals, while significant carotid artery stenosis (CAS) affects approximately 3% of adults over 45 years old, increasing prevalence to 10% in the elderly [29,30,31,32]. CAS resulting in a significant lumen reduction (>50%) is observed in between 10% and 20% cases of all ischemic strokes [30,31,32,33]. It is commonly believed that atherosclerosis develops in parallel manner in diverse arterial territories, including the aorta, coronary, supra-aortic, renal and lower limb arterial beds [34,35,36]. Furthermore, atherosclerosis burden in one arterial territory could predict atherosclerosis extent in another, as well as the risk of adverse cardiovascular events [37,38,39].

To date, the prevalence of carotid plaques and stenosis in patients with FH has not been systematically established, despite the fact that carotid plaque is a strong predictor of MACCE [28,40]. Taking into consideration that carotid intima-media and carotid plaques are commonly recognized as mirrors for atherosclerotic burden in other arterial territories, the lack of consistency in reports puts this hypothesis at risk in patients with FH [41,42,43,44].

Therefore, the aim of this systematic review and meta-analysis was to evaluate the carotid atherosclerosis burden in patients with either clinical or genetic FH in order to establish a potential connection between carotid and coronary atherosclerosis in patients with FH.

## 2. Materials and Methods

This systematic review and meta-analysis were conducted in accordance with the PRISMA 2020 guidelines [45]. The PRISMA 2020 checklist is enclosed in the Supplementary Material (Figure S1) [45].

### 2.1. Eligibility Criteria

We included peer-reviewed studies that reported the prevalence of carotid atherosclerotic plaques and/or stenosis in individuals with either a clinical or genetic diagnosis of FH. Eligible studies enrolled adult participants (≥18 years) diagnosed with FH and provided original data enabling the estimation of carotid plaques/stenosis frequency. Carotid atherosclerotic plaques were required to be assessed using ultrasonography and defined according to the Mannheim consensus criteria: (1) a focal structure encroaching into the arterial lumen by at least 0.5 mm, (2) a lesion protruding ≥50% beyond the surrounding intima–media thickness (IMT), or (3) a focal thickening >1.5 mm measured from the media–adventitia interface to the intima–lumen surface [46]. Studies applying any single criterion consistent with the Mannheim definition were also considered eligible. Carotid atherosclerotic stenosis had to be defined as ≥50% narrowing of the extracranial carotid artery (internal or common), with a specified method of detection, including ultrasonography, computed tomography angiography, magnetic resonance angiography or digital subtraction angiography [47]. In both research and clinical practice, ultrasound is a sufficient and preferred method for assessing the presence of carotid plaques; in contrast, the evaluation of carotid stenosis requires more precise quantification of the degree of narrowing, which explains the broader range of included imaging modalities. Diagnostic criteria for FH were required to be clearly defined, based on either genetic testing or established clinical algorithms, including the DLCN, Simon Broome Register, MEDPED, Japanese Atherosclerosis Society guidelines, Canadian FH criteria, or other established clinical criteria. Alternatively, diagnosis could be based on total cholesterol or LDL-C cutoffs, provided these were combined with additional clinical information, such as personal history of premature ASCVD, family history of hypercholesterolemia or premature ASCVD, and relevant physical findings (e.g., tendon xanthomas). Eligible studies had to provide sufficient data on number of FH individuals, and the number or percentage of individuals with carotid atherosclerotic plaques and/or stenosis. Exclusion criteria comprised case reports, case series with fewer than 10 participants, reviews, editorials, studies lacking sufficient data, and those focused exclusively on homozygous FH. Only full-text articles published in English were included.

### 2.2. Information Sources and Search Strategy

A comprehensive search of PubMed (MEDLINE), Embase, and Scopus was conducted to 3 August 2025. The core search query applied across all databases was: (“familial hypercholesterolemia” OR “FH”) AND (“carotid atherosclerosis” OR “carotid plaque” OR “carotid stenosis”). In addition, database-specific controlled vocabulary was incorporated: for PubMed, “Hyperlipoproteinemia Type II” [Mesh] was used in the first part of the query and “Carotid Artery Diseases” [Mesh] in the second part; for Embase, the corresponding Emtree terms ‘familial hypercholesterolemia’/exp and ‘carotid artery disease’/exp were added. In Scopus, the search was restricted to titles and abstracts due to the high number of records initially retrieved (6244 records without restrictions). Reference lists of all eligible studies and relevant reviews were also screened to identify additional articles. No restrictions on language or publication date were applied.

### 2.3. Selection and Data Collection Processes

**Figure 1:**
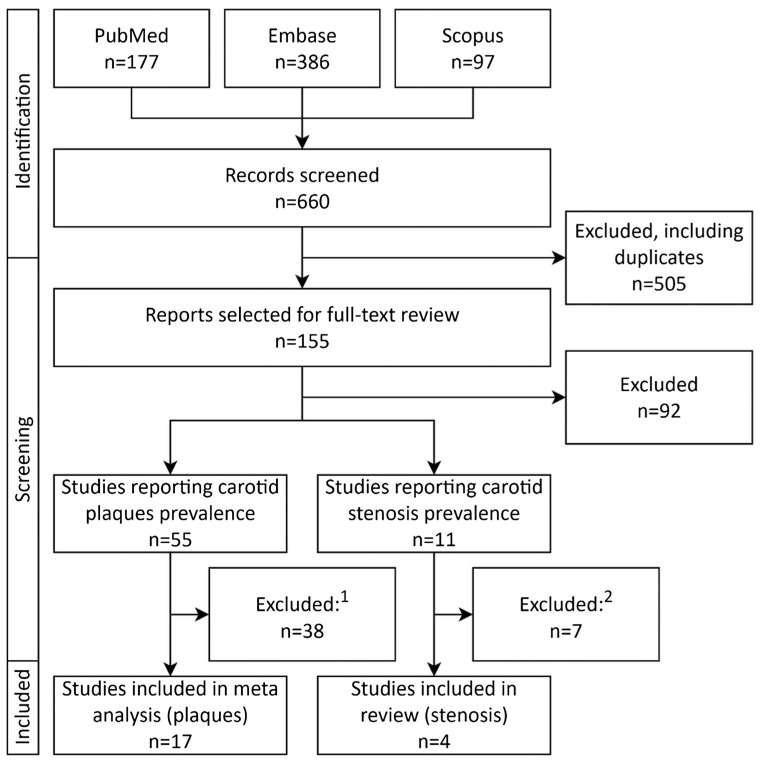
PRISMA study selection flowchart depicting selection and inclusion process of studies. ^1^—Reasons for study exclusion after full-text review: lack of carotid plaque definition (*n* = 9); carotid plaque definition differing from the methodology adopted in this meta analysis (*n* = 7); conference abstract (*n* = 6); study population entirely or partly <18 years old (*n* = 4); overlapping study population with an included study (*n* = 3); full text in a non-English language (*n* = 2); full text not accessible (*n* = 2); multiple reasons (*n* = 5). ^2^—Reasons for study exclusion after full-text review: conference abstract (*n* = 3); full text available in a non-English language (*n* = 2); overlapping study population with an included study (*n* = 1); lack of information regarding carotid stenosis assessment (*n* = 1). Abbreviations: FH—familial hypercholesterolemia.

Titles and abstracts were screened, and the full texts of potentially eligible studies were subsequently assessed by MP. Given the heterogeneity of the included studies and the fact that carotid plaques/stenosis prevalence was often reported as part of population characteristics rather than as a primary study objective, methodological quality was assessed using the Mixed Methods Appraisal Tool (MMAT) [48]. Studies were categorized into two quality groups based on the proportion of MMAT criteria met: high quality (≥80% of criteria fulfilled) and low quality (<80% of criteria fulfilled). Data extraction was performed by MP using a standardized form. Extracted data included study characteristics (author, year, country, DOI), details of carotid artery assessment (plaque definition, imaging modality used for carotid stenosis detection), and population characteristics (sample size, mean or median age, sex distribution, comorbidities including hypertension, diabetes and CAD, mean or median LDL-C concentration, and number of participants with carotid plaques/stenosis). The study selection flowchart is shown in Figure 1.

### 2.4. Data Items and Effect Measure

The primary outcomes were the prevalence of carotid atherosclerotic plaques and carotid stenosis in the adult FH population. Additional analyses evaluated plaque prevalence in studies based on a clinical diagnosis of FH (including those with a subset of genetically confirmed cases), in studies enrolling exclusively genetically confirmed FH individuals, and according to whether participants with established ASCVD were included. For each study, prevalence was defined as the proportion of FH participants presenting with carotid plaques or stenosis. The effect measure used for quantitative synthesis was the pooled prevalence with corresponding 95% confidence intervals (CIs). Additional qualitative data were extracted as counts with percentages, whereas quantitative data were extracted as means with 95% CIs or as medians with the first and third quartiles (Q1–Q3) or interquartile ranges (IQR).

### 2.5. Synthesis Methods

Data were analyzed using R software (version 4.5.0) within the RStudio environment (version 2025.05.0+496). The quantitative and qualitative characteristics of participants in the included studies were summarized using weighted estimates. For studies reporting quantitative variables as median and interquartile range, the Box–Cox method was applied to approximate the corresponding mean and standard deviation. Pooled prevalence estimates were calculated using the inverse-variance method on the Freeman–Tukey double-arcsine transformed proportions. Between-study variance (τ^2^) was estimated using the Restricted Maximum Likelihood (REML) method, and confidence intervals for τ^2^/τ were calculated using the Q-Profile method. Heterogeneity was assessed with Cochran’s Q and I^2^ (based on Q). CI for the pooled estimates were computed using the Hartung–Knapp adjustment, and prediction intervals were calculated to estimate the expected range of prevalence in future studies. A 95% CI for individual study proportions was calculated using the Clopper–Pearson exact method. Predefined subgroup analyses were performed according to method of FH diagnosis and inclusion/exclusion of participants with established ASCVD. Publication bias was assessed through visual inspection of a Doi plot with the Luis Furuya-Kanamori (LFK) index. Results were presented as pooled prevalence with 95% confidence intervals and visualized using forest plots. Sensitivity analyses used a leave-one-out approach, excluding studies with extreme prevalence values to assess their impact on the pooled estimates. The overall certainty of the pooled prevalence estimates was qualitatively assessed using the GRADE approach, considering the methodological quality of the included studies, heterogeneity, and consistency of results across analyses.

## 3. Results

### 3.1. Carotid Plaques

#### 3.1.1. Description of Included Studies

Seventeen international studies with total of 2870 patients enrolled were included in the final analysis, as shown in the PRISMA flowchart depicted in Figure 1. All studies were single-center, observational studies [28,49,50,51,52,53,54,55,56,57,58,59,60,61,62,63,64]. Eleven studies were from Europe [28,50,51,53,55,56,59,60,61,62,64], and six from Central or East Asia [49,52,54,57,58,63]. All seventeen studies on FH reported the prevalence of carotid atherosclerotic plaques defined according to the Mannheim criteria [28,49,50,51,52,53,54,55,56,57,58,59,60,61,62,63,64]. Eight studies fully applied the Mannheim criteria for plaque definition [28,49,52,53,54,57,60,61], while the remaining nine applied them only partially [50,51,55,56,58,59,62,63,64]. Among the included studies, seven enrolled only individuals without a prior diagnosis of ASCVD [49,51,55,56,59,61,62], whereas the remaining ten imposed no such restriction [28,50,52,53,54,57,58,60,63,64]. Five studies diagnosed FH using both DLCN criteria and genetic testing [49,53,54,57,59], with two of them additionally reporting data for genetically confirmed subgroups [53,54]. Four studies included only patients with genetically verified FH [51,52,56,62]. Two studies applied genetic testing in combination with the Simon Broome criteria [50,60], one used genetic testing or defined clinical criteria [28], one genetic testing or the van Aalst-Cohen criteria [61], and another genetic testing or either DLCN or Simon Broome criteria [55]. One study relied exclusively on DLCN [58], while two applied only defined clinical diagnostic criteria [63,64].

Overall, 14 of the 17 studies incorporated genetically confirmed FH cases to varying extents, with reported proportions ranging from 24.9% to 100%. Participant characteristics also varied. The mean or median age ranged from 37 to 56.9 years, with a weighted mean (WM) 47.2 ± 13.4 years. The proportion of men varied between 35.1% and 58.3%, with a weighted proportion (WP) of 47.3%. The prevalence of hypertension ranged from 8.3% to 66.7% (WP: 24.2%), diabetes mellitus from 0% to 15.3% (WP: 4.9%), and coronary artery disease from 0% to 66.9% (WP 13.4%). Reported mean or median LDL-C levels ranged from 127.6 to 363.5 mg/dL (WM: 216.0 ± 73.4 mg/dL).

Details regarding included studies and patient characteristics across individual studies are provided in Table 1.

#### 3.1.2. Prevalence of Carotid Plaques in All Included Studies

**Figure 2:**
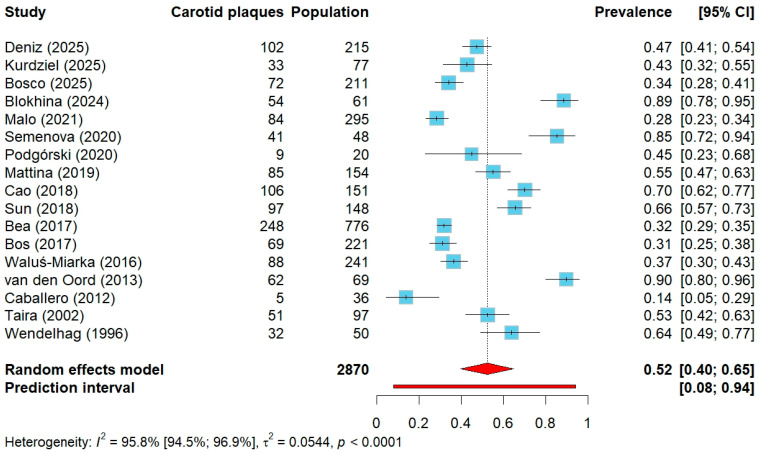
Forest plot of carotid plaque prevalence in all included studies. Abbreviations: CI—confidence interval.

The pooled prevalence of carotid atherosclerotic plaques among 2870 subjects with either clinical or genetic FH was 52% with 95% CI ranging from 40% to 65% (Figure 2). However, heterogeneity was considerable, yielding a wide prediction interval. In the subgroup analysis according to ASCVD status, studies including patients irrespective of ASCVD reported a pooled prevalence of 57% among 1944 individuals (95% CI: 40–73%), whereas in studies restricted to patients without previously established ASCVD, including 926 individuals, the prevalence was 46% (95% CI: 23–70%). In both subgroups, heterogeneity remained very high and the prediction intervals were non-conclusive (Figure S1).

#### 3.1.3. Prevalence of Carotid Plaques in Studies with Clinical Diagnosis of FH (Including Those with a Subset of Genetically Confirmed Cases)

**Figure 3:**
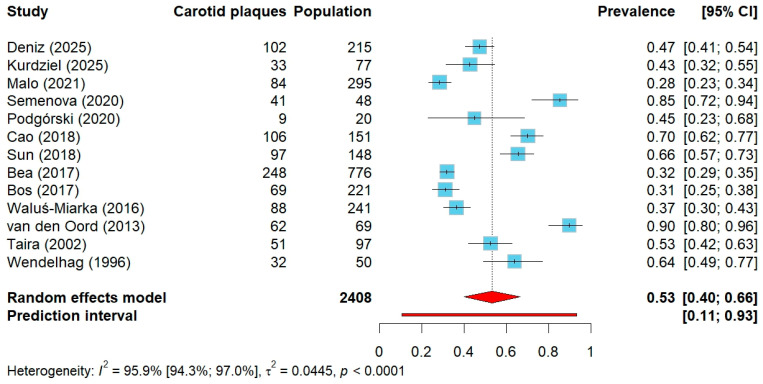
Forest plot of carotid plaque prevalence in studies with clinical diagnosis of FH (including those with a subset of genetically confirmed cases). Abbreviations: CI—confidence interval.

The analysis limited to studies based on a clinical diagnosis of FH, including those with a subset of genetically confirmed cases, encompassed 13 studies with a total of 2408 participants [28,49,50,53,54,55,57,58,59,60,61,63,64]. The proportion of genetically confirmed FH within these studies ranged from 0% (no genetic testing was performed) to 87.4%. The pooled prevalence of carotid plaques in this group was 53% (95% CI: 40–66%), (Figure 3). In studies restricted to participants without previously established ASCVD, the prevalence reached 55% (95% CI: 13–93%), whereas in studies without this restriction it was 53% (95% CI: 37–68%), (Figure S2).

#### 3.1.4. Prevalence of Carotid Plaques in Studies Including Only Genetically Confirmed FH

**Figure 4:**
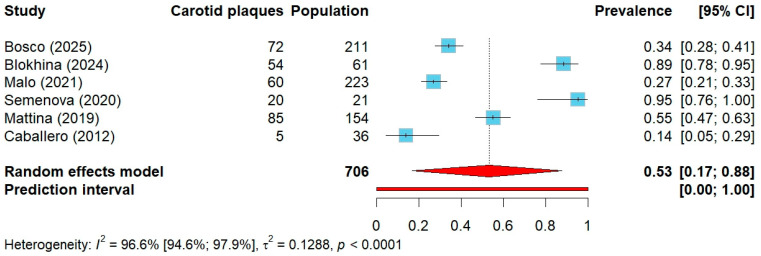
Forest plot of carotid plaque prevalence in studies including only genetically confirmed FH. Abbreviations: CI—confidence interval.

Among 706 individuals with genetically confirmed FH included in 6 studies [51,52,53,54,56,62], the pooled prevalence of carotid atherosclerotic plaques was 53% (95% CI: 17–88%), (Figure 4). When stratified by ASCVD status, studies enrolling patients regardless of ASCVD showed a prevalence of 73% across 305 participants (95% CI: 0–100%), while studies limited to ASCVD-free cohorts, comprising 401 participants, demonstrated a prevalence of 34% (95% CI: 0–85%), (Figure S3).

#### 3.1.5. Publication Bias Assessment, Sensitivity Analysis, and Certainty of Evidence

**Figure 5:**
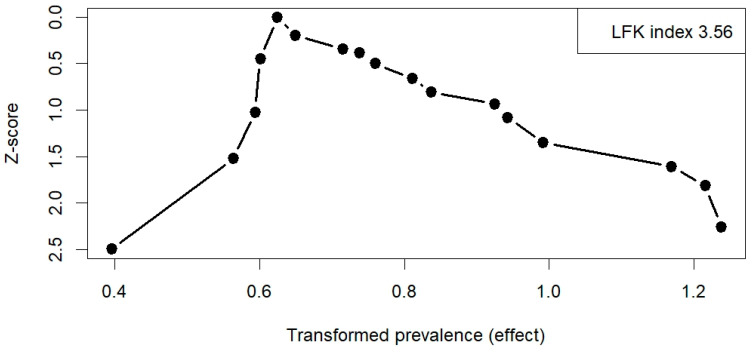
Doi plot of all included studies on carotid plaque prevalence. Abbreviations: LFK—Luis Furuya-Kanamori index.

Across all included studies, the Doi plot demonstrated marked asymmetry, with an LFK index of 3.56, indicating very high heterogeneity among the studies and a strong suggestion of potential publication bias (Figure 5). Similar results were obtained in the subgroup analyses, with an LFK index of 3.73 for studies based on a clinical diagnosis of FH and 2.9 for those including only genetically confirmed FH (Figure S4). Sensitivity analyses, excluding studies with extreme prevalence values, showed broadly similar pooled estimates, suggesting that the results were generally robust. The certainty of evidence for the pooled prevalence estimates of carotid plaques, including subgroup analyses, was rated as low to moderate.

### 3.2. Carotid Stenosis

#### 3.2.1. Description of Included Studies

A total of four studies reporting the prevalence of carotid artery stenosis among patients with FH were included [65,66,67,68] (Table 2). Two were from Europe [66,67], and two from Japan [65,68]. In all studies, stenosis was assessed using ultrasonography [65,66,67,68]; however, only one study specified the use of the NASCET method [65], while the others did not report the method used to estimate the degree of narrowing [66,67,68]. The threshold defining stenosis varied between studies, ranging from 50% to 70%. Two studies included only patients with genetic FH [66,67], one study applied genetic testing in combination with the Japan Atherosclerosis Society guidelines [65], and one used defined clinical criteria [68].

The mean age was reported only in one study [67]. The proportion of men was between 42.1% and 52.4%; the prevalence of hypertension was between 17.3% and 25.4%; diabetes mellitus was between 0% and 9.5%; and coronary artery disease between 26.8% and 100%.

Details of the studies and individuals included in the carotid stenosis prevalence analysis are presented in Table 2.

#### 3.2.2. Prevalence of Carotid Stenosis

**Figure 6:**
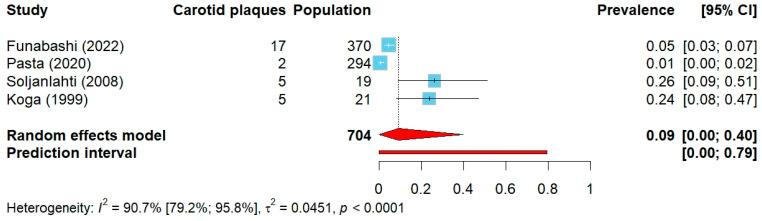
Forest plot of carotid stenosis prevalence. Abbreviations: CI—confidence interval.

The reported prevalence of carotid artery stenosis varied substantially, ranging from 0.7–4.6% in studies with larger populations to 23.8–26.3% in studies with considerably smaller sample sizes. The pooled prevalence of carotid stenosis among 704 FH individuals in 4 studies was 9% (95% CI: 0–40%), (Figure 6).

#### 3.2.3. Publication Bias Assessment, Sensitivity Analysis, and Certainty of Evidence

The Doi plot shows significant asymmetry, with an LFK index of 4.32 (Figure S5). Sensitivity analyses demonstrated that pooled prevalence estimates varied appreciably with the exclusion of individual studies, highlighting instability of the summary effect. The certainty of evidence for the pooled estimate of carotid artery stenosis prevalence was rated as very low.

## 4. Discussion

This systematic review and meta-analysis provide the first comprehensive synthesis of the prevalence of carotid atherosclerosis burden among adults with FH. Our findings indicate a pooled prevalence of carotid plaques of 57% in a total of 2870 subjects, highlighting a substantial burden of carotid atherosclerosis in this high-risk population. Notably, prevalence was higher in studies including patients with established ASCVD compared to ASCVD-free cohorts (57% vs. 46%), suggesting that coexisting CAD or PAD significantly increases carotid plaque burden.

Similarly, the prevalence of carotid artery stenosis in individuals with FH appears highly variable across studies, ranging from less than 1% to over 25%. This heterogeneity likely reflects differences in study populations, diagnostic criteria, and stenosis thresholds, making it difficult to draw firm conclusions. Compared with the general population, where carotid stenosis is estimated to affect approximately 3% of adults, with a sporadic incidence under the age of 50 years [69], the available data may suggest that FH may confer an elevated risk, but the magnitude of this risk remains unclear. Nonetheless, due to the lack of a direct comparison between FH and non-FH subjects within this study, the obtained results must be interpreted with caution. These findings underscore that carotid stenosis in FH is a poorly investigated area.

Nevertheless, all analyses demonstrated substantial heterogeneity, wide confidence intervals, and inconclusive prediction intervals. These findings likely reflect considerable variability across studies, including differences in FH diagnostic criteria, definitions of carotid plaques, and inclusion criteria based on ASCVD status. Study populations also differed markedly in terms of comorbidities (CAD, hypertension, diabetes mellitus), age distribution, and LDL-C levels. Regarding LDL-C, discrepancies arose due to inconsistent reporting, as studies did not uniformly clarify whether reported values represented the highest recorded level, calculated estimates under ongoing lipid-lowering therapy, or current measurements. Finally, variations in lipid-lowering treatment across cohorts likely further contributed to the observed heterogeneity.

The assessment of carotid atherosclerosis burden holds dual and fundamental clinical significance. On one hand, the presence of plaques in the carotid artery is widely recognized as a powerful, non-invasive surrogate marker of systemic atherosclerosis, serving as a ‘mirror’ for the overall atherosclerotic burden in other crucial vascular beds, including the CAD. On the other hand, these plaques are an independent and robust predictor of ischemic stroke and other MACCE. Consequently, confirmation that a substantial proportion of individuals with FH is affected by carotid atherosclerosis provides a foundation for more focused and clinically relevant future investigations.

Given that the weighted proportion of CAD was 13.4%, and the pooled prevalence of carotid plaques reached 52%, it cannot be concluded that, for FH patients, carotid plaques can be reliably positioned as a singular marker of total atherosclerotic burden. These results suggest substantial variability in the spectrum of atherosclerosis extent in the carotid arterial territory. In particular, in patients with genetically confirmed FH, carotid artery involvement varies tremendously. Specifically, the absence of atherosclerotic lesions in carotid arteries may fail to rule out significant CAD and therefore should not serve as a reliable risk determinant in patients with FH. This finding is highly relevant, as researchers have used carotid plaque presence to score cardiovascular risk, including cardiovascular death, MI, and ischemic stroke [44,70]. In the general population, carotid plaque progression or regression may increase or decrease the risk of adverse cardiovascular outcomes [71,72]. In contrast, in FH patients, such an approach is not yet appropriate, emphasizing the need for more comprehensive and multicenter studies. Additionally, a better reappraisal of FH is required to improve the selection of patients with FH [73]. Ray et al. demonstrated that the incidence of cardiovascular events increased with age and was highest for definite FH and lowest for unlikely FH within each age category. The risk of premature cardiovascular events was significantly higher for both definite and potential FH (HR 4.21, 95% CI 3.69–4.81 and HR 3.46, 95% CI 3.07–3.89, respectively) compared to unlikely FH.

While the prevalence of carotid atherosclerosis in the general population is estimated at approximately 20% [29], our results suggest that FH patients represent a particularly vulnerable group, with prevalence rates two to three times higher. Such a marked difference suggests that atherosclerotic changes may begin early in the course of FH, potentially even before adulthood. Tada et al. and Shibayama et al. have reported that carotid plaques begin to develop in the FH population as early as adolescence [74,75]. These estimates are supported by studies on children with FH, among whom carotid atherosclerotic plaques can already be detected. Earlier studies have shown that the prevalence of carotid plaques in children with FH is approximately 10% [76,77,78]. More recent studies involving pediatric FH populations, likely due to advances in treatment methods, have reported primarily CIMT measurements rather than plaque detection [79,80].

Studies indicate that the prevalence of FH among patients with CAD is up to 20 times higher than in the general population, affecting approximately 1 in 15 individuals (6.7%) [2,3]. In light of the high prevalence of carotid atherosclerosis among FH patients, it is reasonable to expect an elevated prevalence of FH among individuals with carotid atherosclerosis, particularly those with carotid stenosis. Nevertheless, this association remains poorly established, as a review of the literature identified only two studies addressing this specific relationship, both published solely in abstract form, which substantially limits their interpretability. Ijäs et al., in a study involving 500 patients with symptomatic and asymptomatic internal carotid artery stenosis undergoing carotid endarterectomy, found that a probable or definite diagnosis of FH based on DLCN criteria applied to 1.8% of participants. However, genetic testing confirmed the diagnosis in only one individual, yielding a prevalence of 1 in 500 [81]. In another study, Perica et al. reported that a possible diagnosis of FH according to DLCN criteria was observed in 11 out of 52 patients (21.15%) with CAS treated with carotid artery stenting [82].

Carotid atherosclerosis is a strong predictor of future MACCE [28,83], which raises the question of whether individuals with FH are at higher risk of stroke. The evidence on this issue remains inconsistent. Some studies have suggested a higher incidence of stroke among individuals diagnosed with FH [84,85]; however, these observations are primarily based on clinical diagnostic criteria, which tend to overestimate the true prevalence of FH [86]. In contrast, several large observational studies and meta-analyses of genetically confirmed FH cases have failed to demonstrate a significantly elevated risk of ischemic stroke in this population [87,88,89,90,91]. This discrepancy may be explained by the fact that clinical diagnostic criteria often capture patients with existing cardiovascular disease, a strong risk factor for stroke, which may confound the observed association [92,93]. Furthermore, the apparent lack of increased ischemic stroke risk in genetically confirmed FH may reflect important differences in the pathophysiological mechanisms underlying stroke versus myocardial infarction. While coronary artery disease is directly driven by lipid accumulation and plaque rupture within the coronary arteries, ischemic stroke is more heterogeneous in origin, often resulting from embolic events, small vessel occlusion, or cardioembolic sources rather than from extracranial large-vessel atherosclerosis alone [94,95,96].

## 5. Limitations

Several crucial limitations must be acknowledged. Firstly, the search was restricted to English-language publications, which may introduce language bias. Secondly, the search, selection, and data extraction were conducted by a single reviewer. Thirdly, most included studies were not specifically designed to assess carotid artery plaques, with plaque evaluation often reported only as descriptive data rather than a primary objective. Fourthly, over half of the studies were of low methodological quality. Fifthly, the studies were highly heterogeneous, and evidence of publication bias was suggested by Doi plot asymmetry and high LFK index values. Sixthly, sensitivity analyses for carotid stenosis demonstrated instability of the summary effect. Collectively, these factors led to an overall certainty of evidence rated as low to moderate for carotid plaques and very low for carotid stenosis.

## 6. Conclusions

This meta-analysis indicates that carotid atherosclerosis may be highly prevalent in adult patients with FH, especially those with genetically confirmed FH, affecting approximately half of this population. These findings highlight the substantial gap in understanding the relationship between FH and carotid atherosclerosis. Future high-quality studies are warranted to refine prevalence estimates and to further elucidate the interplay between these conditions.

## Figures and Tables

**Table 1:** Characteristics of the studies reporting carotid plaque.

First Author (Year)	Country	FH Diagnosis	ASCVD ^1^ [Yes/No]	Carotid Plaque ^2^	Sample Size n	Males n (%)	Females n (%)	HT n (%)	DM n (%)	CAD n (%)	Age [Years] ^3^	LDL-C ^3,4^ [mg/dL]	Genetic Diagnosis n (%)	Plaque Prevalence n (%)	Quality Assessment
Deniz (2025) [49]	Turkey	genetic, DLCN	No	1, 2, 3	215	79 (36.7%)	136 (63.3%)	89 (41.5%)	29 (13.5%)	0 (0.0%)	54 (43–62)	244 (210–277)	46 (21.4%)	102 (47.4%)	high
Kurdziel (2025) [50]	Poland	genetic, Simon Broome	Yes	2, 3	77	NR	NR	NR	NR	NR	NR	NR	NR	33 (42.9%)	low
Bosco (2025) [51]	Italy	genetic	No	3	211	99 (46.9%)	112 (53.1%)	73 (34.6%)	7 (3.3%)	0 (0.0%)	54.73 ± 7.93	274.76 ± 23.62	211 (100%)	72 (34.1%)	high
Blokhina (2024) [52]	Russia	genetic	Yes	1, 2, 3	61	28 (45.9%)	33 (54.1%)	28 (45.9%)	4 (6.6%)	18 (29.5%)	50 (40–61)	median: 310.5	61 (100%)	54 (88.5%)	low
Malo (2021) [53]	Spain	genetic, DLCN	Yes	1, 2, 3	295	156 (52.9%)	139 (47.1%)	30 (10.2%)	3 (1.0%)	7 (2.4%)	40 (32–49)	243 (206–293.44)	223 (75.6%)	84 (28.5%)	high
Malo ^5^ (2021) [53]	Spain	genetic	Yes	1, 2, 3	223	114 (51.1%)	109 (48.9%)	19 (8.5%)	3 (1.3%)	4 (1.8%)	37 (30–49)	259.65 (216–312.36)	223 (100%)	60 (26.9%)	high
Semenova (2020) [54]	Russia	genetic, DLCN	Yes	1, 2, 3	48	25 (52.1%)	23 (47.9%)	29 (60.4%)	NR	25 (52.1%)	NR	NR	21 (43.8%)	41 (85.4%)	low
Semenova ^5^ (2020) [54]	Russia	genetic	Yes	1, 2, 3	21	11 (52.4%)	10 (47.6%)	14 (66.7%)	NR	13 (61.9%)	48 (42–55)	363.5 (321.0–433.1)	21 (100%)	20 (95.2%)	low
Podgórski (2020) [55]	Poland	genetic, DLCN, Simon Broome	No	2	20	10 (50.0%)	10 (50.0%)	7 (35.0%)	0 (0.0%)	0 (0.0%)	37.5 ± 7.4	217.50 ± 53.47	NR	9 (45.0%)	low
Mattina (2019) [56]	France	genetic	No	3	154	70 (45.5%)	84 (54.5%)	19 (12.3%)	0 (0.0%)	0 (0.0%)	48.3 ± 13.8	176.0 ± 64.6	154 (100%)	85 (55.2%)	high
Cao (2018) [57]	China	genetic, DLCN	Yes	1, 2, 3	151	88 (58.3%)	63 (41.7%)	55 (36.4%)	22 (14.6%)	101 (66.9%)	47.21 ± 13.57	217.3 ± 92.0	132 (87.4%)	106 (70.2%)	high
Sun (2018) [58]	China	DLCN	Yes	2, 3	148	77 (52.0%)	71 (48.0%)	53 (36.8%)	22 (15.3%)	87 (58.8%)	49 ± 13	199.2 ± 65.7	NR	97 (65.5%)	low
Bea (2017) [28]	Spain	genetic, clinical	Yes	1, 2, 3	776	367 (47.3%)	409 (52.7%)	130 (27.2%)	18 (2.4%)	74 (9.6%)	46 (35–55)	228 (199–277)	NR	248 (32.0%)	low
Bos (2017) [59]	Netherlands	genetic, DLCN	No	2, 3	221	107 (48.4%)	114 (51.6%)	46 (20.8%)	6 (2.7%)	0 (0.0%)	46 ± 15	127.6 ± 38.7	170 (76.9%)	69 (31.2%)	high
Waluś- Miarka (2016) [60]	Poland	genetic, Simon Broome	Yes	1, 2, 3	241	98 (40.7%)	143 (59.3%)	71 (29.5%)	15 (6.2%)	42 (17.4%)	41 (18.4)	178.3 ± 61.5	60 (24.9%)	88 (36.5%)	high
van den Oord (2013) [61]	Netherlands	genetic, van Aalst-Cohen	No	1, 2, 3	69	36 (52.2%)	33 (47.8%)	11 (15.9%)	2 (2.9%)	0 (0.0%)	55 ± 8	143.1 ± 65.7	19 (27.5%)	62 (89.9%)	high
Caballero (2012) [62]	Spain	genetic	No	2, 3	36	18 (50.0%)	18 (50.0%)	3 (8.3%)	0 (0.0%)	0 (0.0%)	45.7 ± 10.9	171.9 ± 74.8	36 (100%)	5 (13.9%)	low
Taira (2002) [63]	Japan	clinical	Yes	2	97	34 (35.1%)	63 (64.9%)	20 (20.6%)	NR	NR	NR	NR	NR	51 (52.6%)	low
Wendelhag (1996) [64]	Sweden	clinical	Yes	2	50	29 (58.0%)	21 (42.0%)	NR	NR	NR	56.9 ± 12.0	129.2 ± 29.4	NR	32 (64.0%)	low
Weighted proportion/mean						**47.3%**	**52.7%**	**24.2%**	**4.9%**	**13.4%**	**47.2 ± 13.4**	**216.0 ± 73.4 ^5^**			

^1^—Do the study’s inclusion criteria allow for the enrollment of patients with previously established ASCVD? ^2^—definition according to Mannheim Consensus: (1) a focal structure encroaching into the arterial lumen by at least 0.5 mm, (2) a lesion protruding ≥50% of the surrounding intima–media thickness (IMT), or (3) a focal thickening >1.5 mm measured from the media–adventitia interface to the intima–lumen surface. ^3^—data presented as mean ± SD or median (Q1–Q3)/(IQR). ^4^—in cases where LDL cholesterol values were reported in mmol/L, they were converted to mg/dL by multiplying by 38.67. ^5^—data for the subgroup with genetically confirmed FH. Abbreviations: ASCVD—atherosclerotic cardiovascular disease; CAD—coronary artery disease; DM—diabetes mellitus; FH—familial hypercholesterolemia; HT—hypertension; LDL-C—low-density lipoprotein cholesterol; NR—not reported.

**Table 2:** Characteristics of the studies reporting carotid stenosis.

First Author (Year)	Country	FH Diagnosis	Stenosis Diagnosis	Stenosis Cut-Off	Sample Size n	Males n (%)	Females n (%)	HT n (%)	DM n (%)	CAD n (%)	Age [years] ^1^	LDL-C ^1,2^ [mg/dL]	Genetic Diagnosis n (%)	Stenosis Prevalence n (%)	Quality Assessment
Funabashi (2022) [65]	Japan	genetic, JAS	ultrasound, NASCET	>50%	370	159 (43.0%)	211 (57.0%)	94 (25.4%)	9 (2.4%)	99 (26.8%)	NR	NR	260 (70.3%)	17 (4.6%)	high
Pasta (2020) [66]	Italy	genetic	ultrasound	≥60%	294	130 (44.2%)	164 (55.8%)	51 (17.3%)	NR	NR	NR	293.6 ± 71.6	294 (100%)	2 (0.7%)	low
Soljanlahti (2008) [67]	Finland	genetic	ultrasound	≥70%	19	8 (42.1%)	11 (57.9%)	NR	0 (0.0%)	19 (100%)	57.9 ± 3.7	158.5 ± 34.8	19 (100%)	5 (26.3%)	low
Koga (1999) [68]	Japan	clinical	ultrasound	≥50%	21	11 (52.4%)	10 (47.6%)	5 (23.8%)	2 (9.5%)	20 (95.2%)	NR	NR	0 (0.0%)	5 (23.8%)	low

^1^—data presented as mean ± SD. ^2^—in cases where LDL cholesterol values were reported in mmol/L, they were converted to mg/dL by multiplying by 38.67. Abbreviations: ASCVD—atherosclerotic cardiovascular disease; CAD—coronary artery disease; DM—diabetes mellitus; FH—familial hypercholesterolemia; HT—hypertension; JAS—Japan Atherosclerosis Society guidelines; LDL-C—low-density lipoprotein cholesterol; NASCET—North American Symptomatic Carotid Endarterectomy Trial.

## Data Availability

No new data were created or analyzed in this study. Data sharing is not applicable to this article.

## Supplementary Materials

The following supporting information can be downloaded at: https://www.mdpi.com/article/10.3390/jcm14248676/s1, PRISMA 2020 checklist; Figure S1: Prevalence of carotid atherosclerotic plaques in all included studies stratified by ASCVD status; Figure S2: Prevalence of carotid plaques in studies with clinical diagnosis of FH (including those with a subset of genetically confirmed cases), stratified by ASCVD status; Figure S3: Prevalence of carotid plaques in studies including only genetically confirmed FH, stratified by ASCVD status; Figure S4: DOI plots of studies included in the subgroup analysis, stratified according to the diagnostic method of FH; Figure S5: Doi plot of the studies reporting carotid stenosis.
