# Supplementary material for: Prevalence of Carotid Atherosclerotic Plaques and Stenosis in Adults with Familial Hypercholesterolemia Needs Reappraisal: Systematic Review and Meta-Analysis
Source: J Clin Med. 2025 Dec 7;14(24):8676. doi: 10.3390/jcm14248676 (PMC12733589; doi:10.3390/jcm14248676)

Figure S1. Prevalence of carotid atherosclerotic plaques in all included studies stratified by ASCVD status

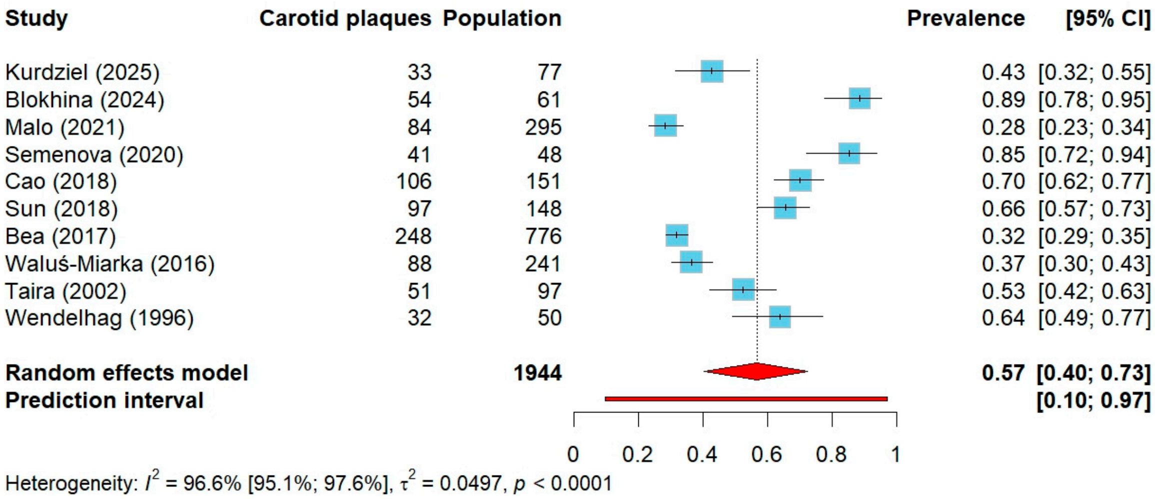

A. Studies including patients irrespective of ASCVD status

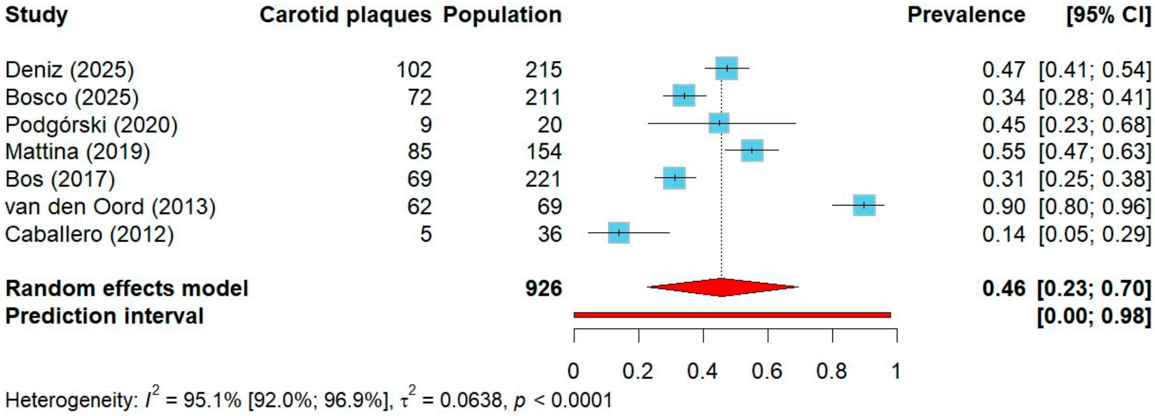

B. Studies including only patients without established ASCVD

Figure S2. Prevalence of carotid plaques in studies with clinical diagnosis of FH (including those with a subset of genetically confirmed cases), stratified by ASCVD status

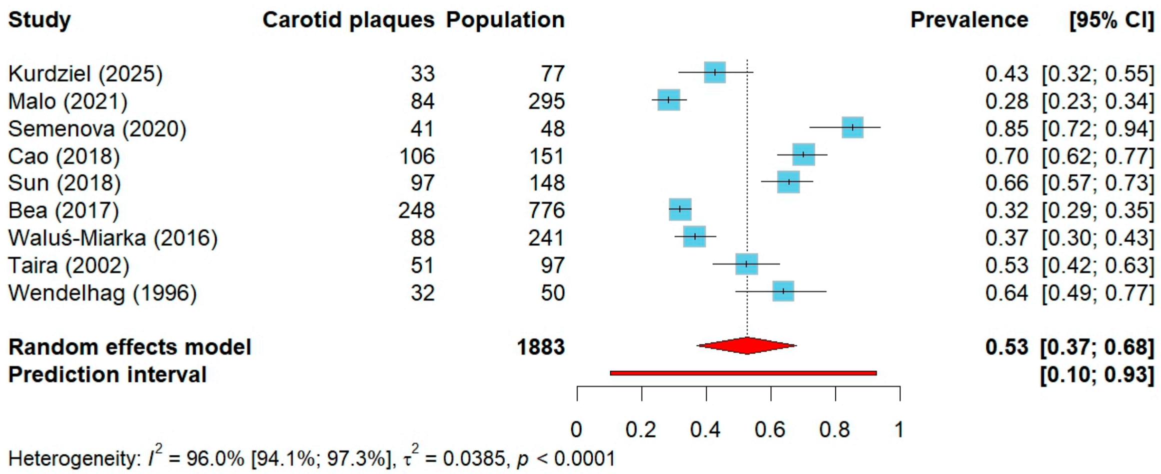

A. Studies including patients irrespective of ASCVD status

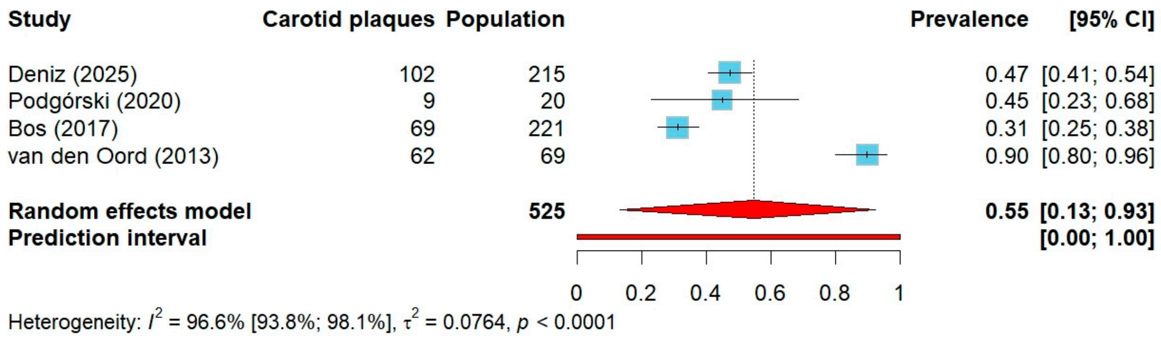

B. Studies including only patients without established ASCVD

Figure S3. Prevalence of carotid plaques in studies including only genetically confirmed FH, stratified by ASCVD status

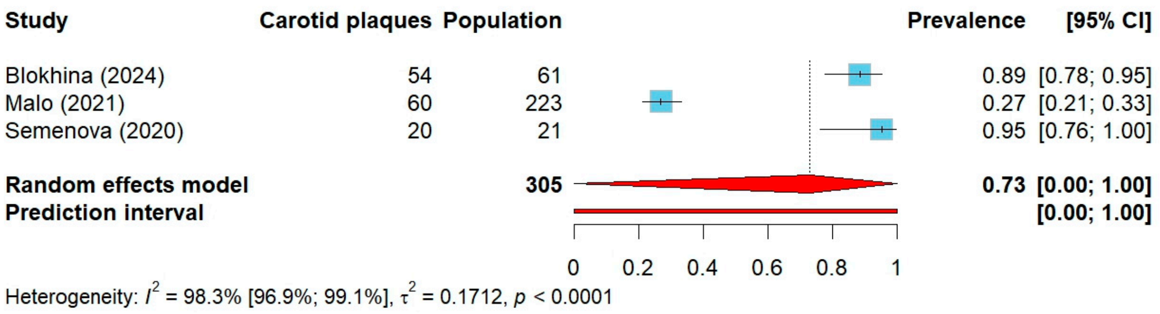

A. Studies including patients irrespective of ASCVD status

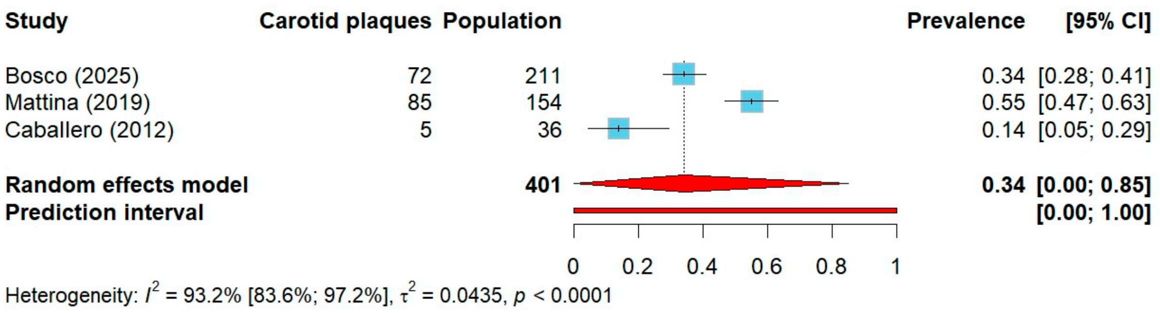

B. Studies including only patients without established ASCVD

Figure S4. DOI plots of studies included in the subgroup analysis, stratified according to the diagnostic method of FH

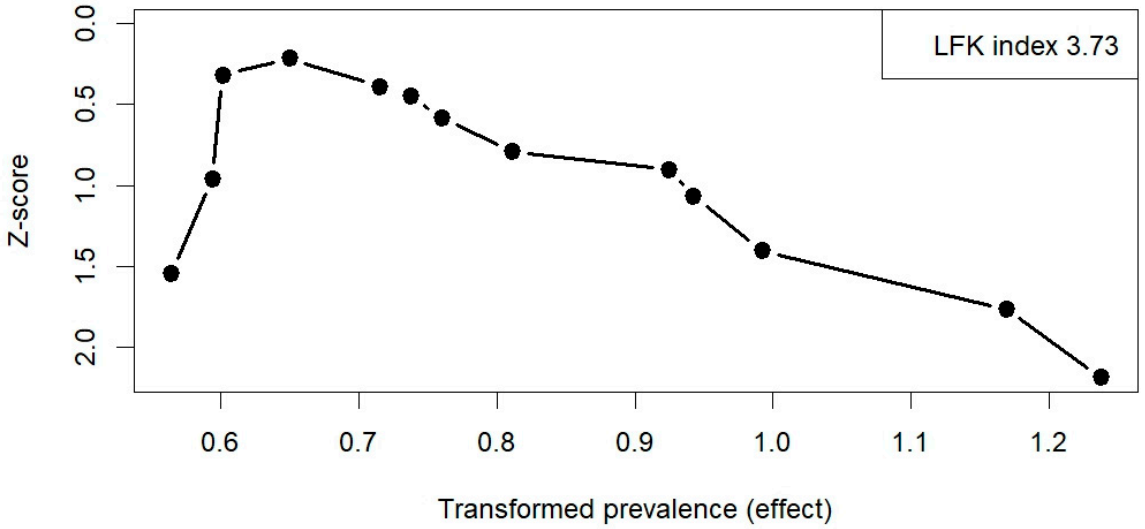

A. with clinical diagnosis of FH (including those with a subset of genetically confirmed cases)

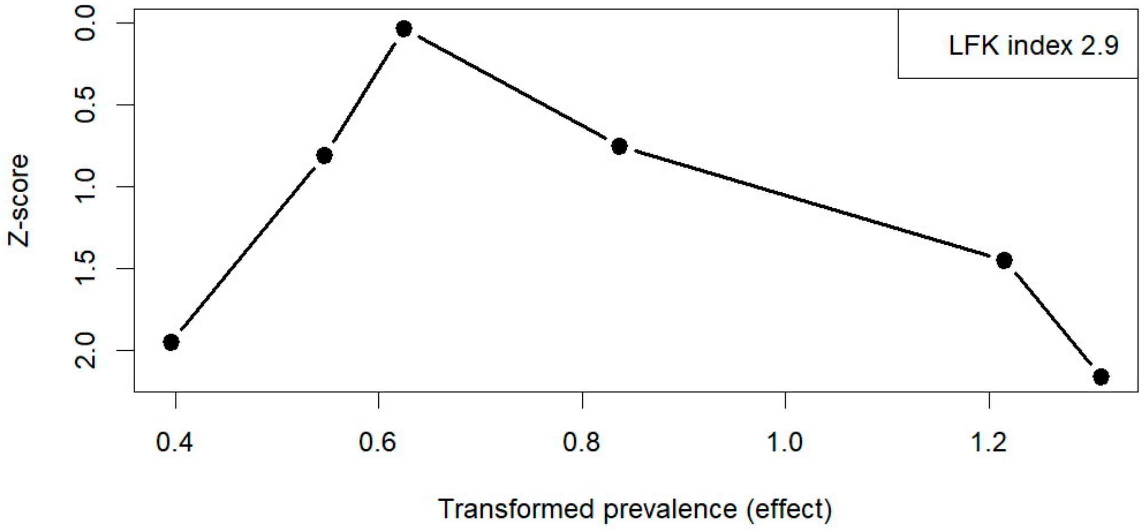

B. Studies including only genetically confirmed FH

Figure S5. Doi plot of the studies reporting carotid stenosis

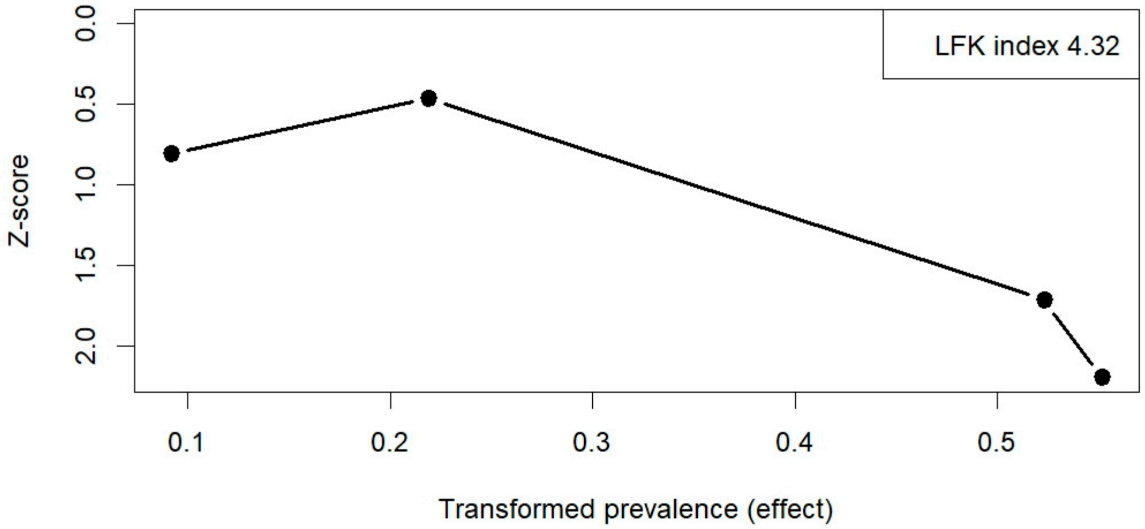

Supplement: Supplementary file 1 [file jcm-14-08676-s001.zip › jcm-4003531-supplementary 2 02.12.2025.pdf]
